# LADES: A Software for Constructing and Analyzing Longitudinal Designs in Biomedical Research

**DOI:** 10.1371/journal.pone.0100570

**Published:** 2014-07-01

**Authors:** Alan Vázquez-Alcocer, Daniel Ladislao Garzón-Cortes, Rosa María Sánchez-Casas

**Affiliations:** 1 Centro de Investigación en Matemáticas, Guanajuato, Guanajuato, México; 2 Universidad Autónoma de Nuevo León, Facultad de Medicina Veterinaria y Zootecnica, Escobedo, Nuevo León, México; 3 Universidad Autónoma de Nuevo León, Centro de Investigación y Desarrollo en Ciencias de la Salud, Monterrey, Nuevo León, México; Queen's University Belfast, United Kingdom

## Abstract

One of the most important steps in biomedical longitudinal studies is choosing a good experimental design that can provide high accuracy in the analysis of results with a minimum sample size. Several methods for constructing efficient longitudinal designs have been developed based on power analysis and the statistical model used for analyzing the final results. However, development of this technology is not available to practitioners through user-friendly software. In this paper we introduce LADES (Longitudinal Analysis and Design of Experiments Software) as an alternative and easy-to-use tool for conducting longitudinal analysis and constructing efficient longitudinal designs. LADES incorporates methods for creating cost-efficient longitudinal designs, unequal longitudinal designs, and simple longitudinal designs. In addition, LADES includes different methods for analyzing longitudinal data such as linear mixed models, generalized estimating equations, among others. A study of European eels is reanalyzed in order to show LADES capabilities. Three treatments contained in three aquariums with five eels each were analyzed. Data were collected from 0 up to the 12th week post treatment for all the eels (complete design). The response under evaluation is sperm volume. A linear mixed model was fitted to the results using LADES. The complete design had a power of 88.7% using 15 eels. With LADES we propose the use of an unequal design with only 14 eels and 89.5% efficiency. LADES was developed as a powerful and simple tool to promote the use of statistical methods for analyzing and creating longitudinal experiments in biomedical research.

## Introduction

Most biomedical research projects are planned according to the number of measures to obtain a desired level of accuracy to test the hypothesis of interest. The number of laboratory animals required depends on different factors such as the available budget to obtain the animals; costs regarding housing, adapting time, and hiring qualified personnel; research procedure time, and cost. However, animal ethics represent the most important ‘cost’ of developing a biomedical study. Thus, one of the most common and critical challenges in biomedical studies involving animal models is the minimum number of individuals needed to achieve a certain statistical power or validity. The National Center for the Replacement, Refinement and Reduction of Animals in Research (NC3Rs) promotes the use of experimental design and statistical analysis as important tools to use as few animals as possible without affecting the efficiency of the study. Therefore, biomedical researchers are always looking for cost-efficient designs, i.e. designs with lower cost and more accuracy as possible in their results. A comprehensive guideline for the design of experiments with laboratory animals is presented in [1]. Moreover, the method used to evaluate efficiency or power factor analysis or other types of experimental designs is described in [2]. The most common approaches for calculating sample size in animal experiments are provided in [3]. However, these methods work only with a univariate approach; i.e. one observation per subject. Such a univariate approach may be useful in some cases; however, most biomedical studies are focused on evaluating the development, for example, of a new drug over time or the effects resulting during a time period of a new treatment. Such studies are special, as the result of the last time-response of an individual influences the next; i.e., within-subject observations are correlated. Longitudinal analysis is very useful when we want a statistical model that includes the correlation between within-subject measures. Popular techniques for analyzing longitudinal data are mixed linear models as noted in [4]. Another option is generalized linear mixed models [5], [6]; also, generalized estimating equations [7], [8], and generalized least squares as described in [9], among others. With regard to the problem of sample size, the approach to assess the number of animals needed for a longitudinal study vary according to the statistical model and methods to be used. For generalized estimating equations, the formulas provided in [10] are very useful. Furthermore, for the mixed linear model under continuous responses, the F-Helms statistic provided in [11] is a direct way to calculate statistical power in longitudinal designs. Under this framework Helms proposed a methodology for creating efficient incomplete designs. This type of designs are very useful in animal experiments, since we can calculate the required sample size under different limitations of the cost of maintaining animals throughout the experiment. Despite these important contributions, the analysis of power for longitudinal experiments is still open to study due to the value of research in more complex statistical models that can be applied to biomedical studies [12], [13], [14], [15]. Advances have been made in relation to the power of statistical computing and the optimal design of the experiments. For example, Reich and colleagues provide a data generating model in a freely available software package (clusterPower) for R where the estimation of statistical power is calculated as a factor in the effect of randomized or cross-group test designs [16]. New biostatistical methods for calculating power and sample sizes for data analysis of a mocrobiome are presented by [17] in the software package HMP: Hypothesis testing and power calculations for comparing metagenomic samples of HMP. However, the use of this code based R package is difficult for biomedical professionals who have no experience with programming commands. Moreover, [18] proposed the construction of an optimal design of experiments to reduce the time and cost of modeling biochemical reaction networks using coupled differential equations; a task that can be done using ModelDiscriminationToolkitGUI. There are other softwares for sample size calculation in longitudinal models. The nQuery [19] includes sample size computations for repeated measures analysis under continuous and binary responses; however, we cannot design longitudinal experiments for more than two groups and it is not free. The PASS 12 commercial software [20] also includes sample size estimation for repeated measures analysis but you can include up to three groups. Nevertheless, it does not include statistical methods to analyze the results once we have collected the data. The Optimal Design Software for Multilevel and Longitudinal Research [21] is a free software that can produce graphical analysis for sample size estimation for a wide variety of social research studies performed in classrooms, schools, communities, clinics, etc. Nonetheless, since this software is only focused on applications in the field of social research, the implementation of its power analysis tools throughout its user interface to studies in other fields such as biomedical or engineering it is not direct and easy.

As a result, we believe that good software must have certain conditions: contain more useful methods for sample size and analysis of data, a nice user interface, flexibility for being used in other kind of studies (e.g. industrial or psychological), and accessibility for most researchers and biomedical professionals. The purpose of this paper is to present LADES (Longitudinal Analysis and Design of Experiments Software) for longitudinal data analysis, and construction of efficient longitudinal designs. LADES provides tools for the construction and analysis of longitudinal experiments using a user-friendly interface. LADES is a free and good alternative for biomedical and medical researchers who are not familiar with specialized statistical methods. In addition, it has been created on the basis that there are not many computer programs whose scope is the creation of longitudinal designs with necessary and appropriate cost efficiency. LADES capacity to build cost-efficient designs is demonstrated by analyzing a study of the European eel in which the response of interest was generated sperm volume.

## Materials and Methods

### Introduction to LADES

LADES includes statistical methods for designing biomedical studies and analyzing their results. Moreover, one of its main objectives is to provide an easy to use interface for biomedical researchers and professionals who have only a basic knowledge of statistical methods. LADES includes a module that allows us to calculate sample size for specific cases of problems of longitudinal data with continuous and/or binary responses. A module also exists for creating a variety of experimental designs and optimal designs based on [22]. LADES provides statistical methods such as generalized estimating equations for modeling covariates like Time and correlation between outcomes; linear mixed models for including fixed and random effects in the model; generalized linear mixed models for analyzing discrete outcomes; and generalized least squares to cope with unequal variances of the observations.

Moreover, LADES has the ability to evaluate and create optimal designs in cost and power, using power analysis and F-Helms statistic. With regard to the importance of these issues, LADES is an excellent alternative for the analysis and evaluation of the longitudinal design of experiments for biomedical studies.

LADES was built using JAVA programming language and all functions are built into R [23]. LADES uses numerous R packages including nlme [24], ggplot2 [25], AlgDesign [26], FrF2 [27], and geepack [28]. LADES is available in its project home page: http://cimat.mx/∼hectorhdez/lades/index.html. It runs on Windows 7 and Windows 8 operating systems.

In the following sections some of the capabilities of LADES using real data will be presented. All information about the installation of the software, how to run it and how to perform the calculations showed in the following sections can be found in the Manual available in the More Information section in LADES’ home page.

### Problem

The information provided is from a study by [29] where the amount of sperm produced by European eels was evaluated. The main purpose of this software is to build a longitudinal design optimizing the number of eels, and providing a reasonable power to test the difference between the slopes of three treatments. Biomedical properties are described as follows. Three aquariums containing five eels each were filled with three different treatments (A, B, and C). Thirteen measurements were made over time; one on the day treatment was started and during the remaining twelve weeks post treatment. Therefore, the experimental design was completely longitudinal with 15 eels and 13 measures over time. The response in this study was the sperm volume (ml 100 g fish -1) from each eel. Now, the entire procedure to obtain an efficient longitudinal design for comparing the slopes of the three treatments is shown.

#### Plotting

**Figure 1:**
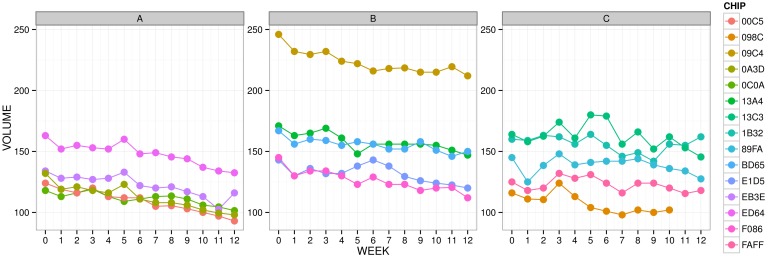
Sperm volume profiles for European Eels grouped by Treatment. CHIP variable was used to indicate each eel. Eel 098C did not present sperm volume in the last two weeks.

In order to obtain an overview of the resulting sperm volume profiles for each eel under study, the Longitudinal Data Graph function in LADES was used. Figure 1 depicts the sperm volume profiles for European eels grouped by Treatment. Differences between the starting points of each eel can be seen in the graph; therefore, a linear mixed model with a random intercept to model these discrepancies was used. The random intercept helps us to model variations due to differences (e.g., genetics), among eels. Moreover, Figure 1 provides insights about differences among the average slopes of the three groups.

#### Fitting the Statistical Model

The proposed data sperm production of European eel statistical model is:
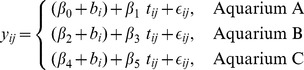
(1)where 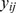 represents the 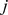-ith measurement of the 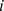-th eel sperm production. The parameters 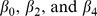 represent the average response at the time of treatment, whereas 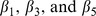 represent the average slopes for the Aquarium A, Aquarium B and Aquarium C, respectively. 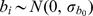 is the random factor for representing the difference between the initial volume of sperm of the eels and the overall mean (as indicated in the previous section); and 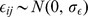 the model residuals. Moreover, 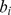 and 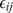 are independent of each other. We use this model because it leads us to more direct comparison of the slopes of the three treatments.

We fitted the linear mixed model 1 using Restricted Maximum Likelihood [30], which is the method for estimating the parameters of interest. The results of the Linear Mixed Model function in LADES for the estimated parameters were
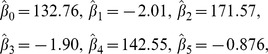


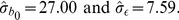
(2)


#### Evaluating Power and Cost

We want to compare the evolution over time of sperm volume of the three aquariums. Therefore, the hypothesis was based on the mean of the slopes of the three treatments. This hypothesis to test is shown below:
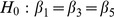
(3)


using an alpha of 0.05. In order to test the hypothesis, F-Helms statistic is used.

LADES assess the cost of a longitudinal design using the following simple formula:
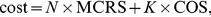
(4)where 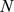 is the total number of subjects, 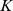 is the total number of observations per subjects, MCRS is the marginal cost of inclusion (e.g., purchase) of an individual in the study, and COS is the cost of evaluating one individual on one occasion. For this problem, we use the values 70 and 3.3 Euros for MCRS and COS, respectively. Such values are raw estimates (according to research experience) of the true cost of experimentation and price of a single European eel and are used just to exemplify software capacities. These values represent the case when cost of acquiring an individual is too high compared to the cost of obtaining an observation.

Using the Longitudinal Design Evaluate function, we can calculate the power the designs provide to test 3, and the cost of the design. This feature also allowed us to evaluate intentionally incomplete longitudinal designs proposed by Helms. The number and label of the steps in time, the parameter estimates shown in 2, and the contrasts of interest (shown in 5) are necessary in the function. The result of this function is the p-value based on 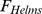 statistic, the power of design, and the total cost of the design. The full original design had a 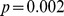, so there is a significant difference between the means of the slopes of the three aquariums. The design showed 88.7% power to test the proposed hypothesis and the design had a total cost of 1693.5 Euros.
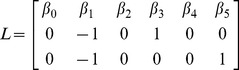
(5)


#### Creating Unequal Longitudinal Designs

Unequal longitudinal designs (ULD) are based on assigning different individuals to groups in order to obtain the distribution that maximizes the power of the test under study. For our problem we can define such designs as follows:
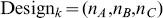
(6)where 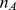, 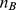 and 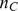 are the number of eels in Aquarium A, B and C, respectively. Two constrains are established and described as follows:

1. 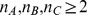. At least 2 eels per group, according to researchers experience; and,
2. 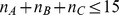. Designs using less than 15 eels (full design) are desired.

**Figure 2:**
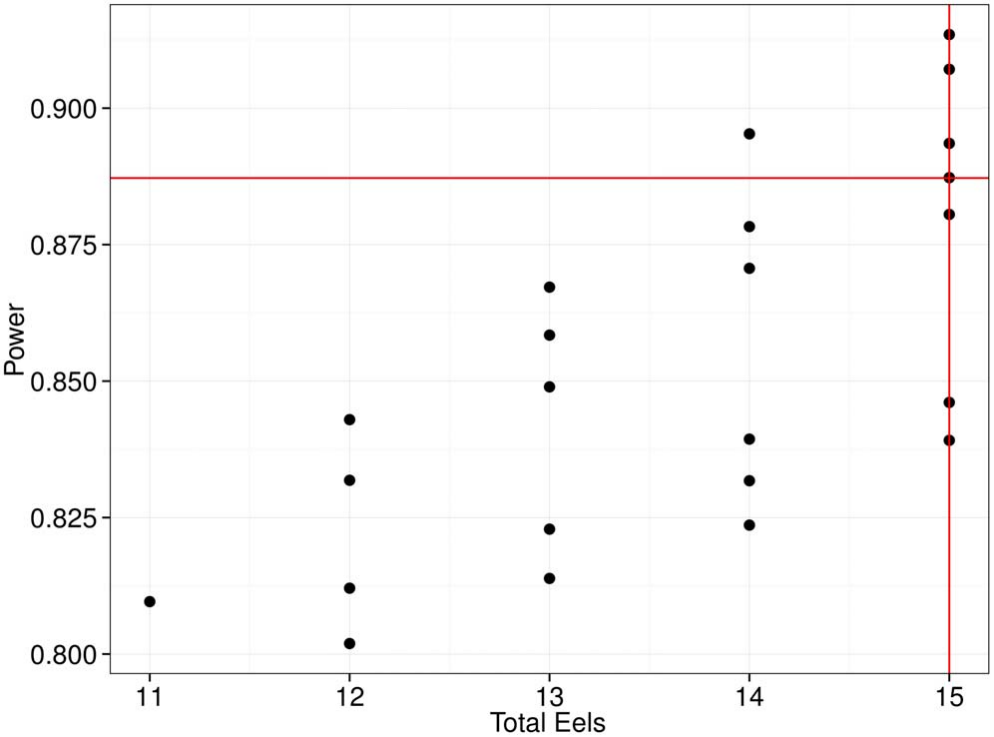
Unequal Cost-Efficient Designs for the European Eel Study. Red lines represent the total number of eels (15) and power (0.887) of the original longitudinal design. Four designs using fifteen eels or less are more powerful than the original design.

The Unequal Longitudinal Designs function in LADES generates unequal designs. This function requires the parameter estimates, the hypothesis (contrasts), the power, and cost of the original design that will be compared to the new designs, as inputs. The results generated by this function for the eel study are: ULD Design Graph: 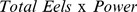 graph where all designs requiring less total number of eels and with a power greater than 0.8 (as it has been shown to be a convention for ‘high power’, [31, page 56], [32]) are depicted (see Figure 2). ULD Designs: presents a table where we can see all the characteristics of the constructed cost-efficient designs (Table 1).

**Table 1:** Cost Efficient Designs.

Design	Aquarium A	Aquarium B	Aquarium C	N Eels	Power	Total Measures	Total Cost
1	5	4	6	15	0.913	195	1693.5
2	4	5	6	15	0.907	195	1693.5
3	4	4	6	14	0.895	182	1580.6
4	6	4	5	15	0.894	195	1693.5
5	5	5	5	15	0.887	195	1693.5
6	4	6	5	15	0.881	195	1693.5
7	5	4	5	14	0.878	182	1580.6
8	4	5	5	14	0.871	182	1580.6
9	5	3	5	13	0.867	169	1467.7
10	4	4	5	13	0.858	169	1467.7
11	3	5	5	13	0.849	169	1467.7
12	6	5	4	15	0.846	195	1693.5
13	4	3	5	12	0.843	156	1354.8
14	6	4	4	14	0.839	182	1580.6
15	5	6	4	15	0.839	195	1693.5
16	3	4	5	12	0.832	156	1354.8
17	5	5	4	14	0.832	182	1580.6
18	4	6	4	14	0.824	182	1580.6
19	5	4	4	13	0.823	169	1467.7
20	4	5	4	13	0.814	169	1467.7
21	5	3	4	12	0.812	156	1354.8
22	3	3	5	11	0.81	143	1241.9
23	4	4	4	12	0.802	156	1354.8

Full Information of the cost-efficient designs for the European eel experiment.

In Figure 2 we can detect an experimental design having more power than the original design (red horizontal line) and requiring only fourteen eels.

## Results and Discussion

For ethical, economic and time reasons, it is important to design efficient experiments on the use of animals needed to obtain good power efficiency using the lowest number of individuals to achieve the efficiency targets set by biomedical researchers. In addition, we have enough observations to detect significant effects on the study process. Consistently, researchers and professionals are encouraged to consult a statistician in the design stage, in addition to highlighting the importance of having a clear idea of the true model and statistical analysis used to analyze the resulting data. These guidelines are provided to assist biomedical researchers and professionals to carry out experiments efficiently. LADES supports researchers with this parameter from a statistical point of view in the design of animal experiments. LADES provides correct design of efficient experiments according to the budget and goals of biomedical research and the power efficiency of the statistical tools required. To our knowledge, there is no program like LADES that allows us to use statistical methods to calculate the required sample size, achieving power efficiency in our tests as well as allowing the combination of other factors, such as repetition, the number of samples required, and mainly, costs. In this research, 23 simulations were carried out with LADES. We determined that researchers can perform simulations, playing with the number of replicates, individuals and the costs and other factors, streamlining the design of choice for the study. On the other hand, the power efficiency can be seen from least to most individuals, allowing the researcher to make the decision on the number of samples to work on his/her project. Analysis of the results obtained in LADES and checked in parallel with the actual experiment, are presented in the following order, cost, power efficiency and eel sperm production during the 12 weeks of study (Table 1). In design 1, 2, 4, 5, 6, 12, and 15, different numbers of eels are combined in aquariums A, B, and C. In the actual experiment the number of individuals used was 15 and the total measurements were 195, with a final cost of 1693.50 Euros, but with different effective power from 0.839–0.913. The researcher can choose the same cost, efficiency and power using a different number of individuals. An example is measurement 22 at a cost of 1241.90 Euros and a power of 0.810, with only 143 measurements, this with a savings of 26.7% with respect to a more expensive experiment (1693.50). Other measurements showed results between the above costs, which the investigator can choose based on his/her budget, time, personnel, and other factors. The graph in Figure 2 correlates the number of individuals with the power of the experiment. For example, we can see that with 11 individuals a power of 0.810 would be obtained, if in addition 12 eels were used you would get an efficiency from 0.802–0.843 with 156 measurements. Using 14 individuals, it would range from 0.824–0.895 with 182 measurements. In the research with European eels that was compared to LADES, 15 animals were used, and a power of 0.887 was obtained with 195 measurements. By designing this experiment in LADES with the same number of individuals (15) and the same measurements but with different combinations a power range of 0.839–0.913 was found. Therefore, investigators can definitely play with different combinations of individuals, budgets, and times with a certainty that they will get acceptable power efficiency for their research.

We have to note that the a-priori information for power analysis was obtained by assuming that values in 2 are the true parameter values. Therefore, the resulting designs will only be valid and efficient for new studies involving the same conditions and factors under study. At the designing stage of a new study, the results from pilot studies are very useful and recommended for estimating sample size when no previous information is available.

Future LADES development will focus on including a module for sample size estimation under drop-out and functions for performing robust analysis to designs. The next version will also aim to improve software speed and stability.

## Supporting Information

Dataset S1 **European eel data.** Aquarium refers to treatment. CHIP is the identification number for each eel. Weeks represents time; number of weeks of the study. Volume is the sperm eel volume in ml 100 g fish -1. B0A and B1A are auxiliar columns for estimating the effects of the intercept and average slope of Aquarium A, respectively, under the parametrization proposed in model 1. Same for Aquarium B and C.
(CSV)
